# Rapid Metabolic and Behavioral Maladaptations Following Short‐Term Obesogenic Diet Withdrawal During Postweaning Development in Male Wistar Rats

**DOI:** 10.1002/brb3.71492

**Published:** 2026-07-09

**Authors:** Breno Picin Casagrande, Vitória Rios Beserra, Alessandra Mussi Ribeiro, Luciana Pellegrini Pisani, Debora Estadella

**Affiliations:** ^1^ Biosciences Department, Institute of Health and Society Federal University of São Paulo Santos São Paulo Brazil

**Keywords:** anxiety‐like behavior, hippocampus, inflammation, obesogenic diet, oxidative stress, withdrawal

## Abstract

**Background:**

Obesogenic diets (ODs) are known to trigger metabolic and inflammatory disturbances. However, the effects of short‐term OD withdrawal on systemic and neuroinflammatory parameters remain unclear.

**Objectives:**

This study investigated the short‐term effects of OD withdrawal on metabolic, inflammatory, and anxiety‐like behaviors in young male Wistar rats.

**Methods:**

Three‐week‐old male Wistar rats were fed either a control (Ct, *n* = 5) or high‐sugar/high‐fat (HSHF) diet for 14 days. Animals in the HSHF group were further divided into no‐withdrawal (NWt, *n* = 5) and withdrawal (Wt, *n* = 5) groups, where Wt received a control diet for 48 h. Food intake, body mass, adiposity, serum metabolic parameters, hepatic energy stores, inflammatory markers (serum, liver, hypothalamus, hippocampus, mesenteric fat), and oxidative stress markers in the hippocampus were measured. Elevated plus maze and open field were used to characterize behavioral phenotype.

**Results:**

OD intake significantly increased caloric intake, visceral adiposity, hepatic glycogen, and triacylglycerol (TAG) levels. The 48‐h withdrawal reduced TAG, induced hyperinsulinemia and hypoglycemia, and heightened inflammation in mesenteric fat, serum, and the hippocampus. Oxidative stress markers (superoxide dismutase [SOD] and malondialdehyde [MDA]) increased in the hippocampus, correlating with elevated serum corticosterone and heightened anxiety‐like behavior in the Wt group compared to the other groups.

**Conclusion:**

Short‐term withdrawal after only 2 weeks of OD intake exacerbates systemic and neuroinflammation, hippocampal oxidative stress, and anxiety‐like behaviors, indicating rapid negative responses to dietary transition. These findings identify short‐term dietary withdrawal during early development as a distinct and biologically active phase, rather than an immediate recovery period.

## Graphical abstract

Postweaning male Wistar rats were fed a high‐sugar/high‐fat (HSHF) diet for 14 days, with or without a 48‐h withdrawal period followed by return to the control diet. Continued HSHF intake increased visceral adiposity, hepatic glycogen, and triacylglycerol accumulation, as well as systemic and central inflammatory markers. Short‐term withdrawal disrupted glucose and lipid homeostasis, maintained elevated visceral adiposity, increased corticosterone levels, and increased inflammation in mesenteric fat and hippocampus, together with higher hippocampal oxidative stress. Withdrawal also increased anxiety‐like behavior in the open field and elevated plus maze, along with reduced exploratory and risk‐assessment behaviors. Anxiety‐like behavior was correlated with inflammatory markers (mainly TNFα), corticosterone, and hippocampal oxidative stress. These findings show that abrupt withdrawal from an obesogenic diet during postweaning development induces rapid metabolic, inflammatory, oxidative, and behavioral changes rather than an immediate recovery state.

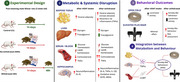

## 1 Introduction

Obesogenic diets (ODs), characterized by high levels of sugar, fat, or a combination of both, are well‐known for their negative effects on several respects of general health (Morris et al. 2015; Afshin et al. 2019). Long‐term improvements in diet can provide significant health benefits, but short‐term metabolic and behavioral consequences of shifting to a healthier diet show mixed results (Morris et al. 2015; Hazarika et al. 2016; Hazarika et al. 2017). Despite the expected general recovery at any point after dietary improvement, it does not immediately or necessarily happen, especially in the short‐term (Casagrande and Estadella 2020; Parnarouskis et al. 2022).

The abrupt removal of energy‐dense, palatable, and ODs can elicit metabolic and behavioral responses that resemble addictive substance withdrawal, including reduced food intake, craving, heightened stress responses, and increased systemic inflammation (Casagrande and Estadella 2020; Parnarouskis et al. 2022; Hough et al. 2026). These responses are thought to arise from metabolic and neuroendocrine shifts, but the precise mechanisms remain unclear. These outcomes are particularly concerning during early life, a critical period for brain development and susceptibility to dietary stressors (Morris et al. 2016; Blanco‐Gandía et al. 2019; Cusick and Georgieff 2016).

Understanding the underlying neurobehavioral mechanisms of OD withdrawal is essential, particularly given its association with anxiety‐like behaviors. Although the nutritional components—sugar, fat, and palatability—do not have drug‐like effects per se, the feeding behavior and its ambience lead to pro‐reward and anti‐stress effects that are self‐reinforcing in a drug‐like manner. Similar patterns are seen in other behaviors, where physiological and behavioral adaptations sustain the habit and contribute to relapse (Soleymani et al. 2016; Gardner et al. 2021; Sliedrecht et al. 2019; Agboola et al. 2010). In the context of ODs, these processes may also shape the response to withdrawal, particularly in the short term.

ODs are known to disrupt metabolic control and affect behavior (Morris et al. 2015; Afshin et al. 2019; de Freitas Queiroz Barros et al. 2026), it increases adiposity and promotes low‐grade inflammation and oxidative stress (Dludla et al. 2019; Rogero and Calder 2018; Tan et al. 2018). These changes contribute to insulin resistance and lipid accumulation in the liver; excess carbohydrate intake can increase de novo lipogenesis and fat deposition (Forrester et al. 2018; Irimia et al. 2017). These effects are partly driven by oxidative stress caused by reactive oxygen species (ROS), which, while essential for cellular signaling in moderate amounts, become damaging when unbalanced (Poprac et al. 2017). Excess ROS leads to lipid peroxidation, protein carbonylation, and irreversible cellular damage, increasing the risk of chronic diseases (Cichoż‐Lach and Michalak 2014). Adipose tissue expansion further increases the risk of insulin resistance, due to the release of free fatty acids, ROS, and inflammatory cytokines (Kawai et al. 2021; Ahmed et al. 2021).

The brain is markedly vulnerable to these disturbances due to its high metabolic demand and limited antioxidant capacity (Salim 2017). Oxidative stress and neuroinflammation activate microglia and astrocytes, leading to sustained cytokine production and disruption of neuronal function (Bondia‐Pons et al. 2012; Simpson and Oliver 2020). Within the brain, the hippocampus is especially sensitive to these insults, while it plays a central role in memory and emotional regulation (Fanselow and Dong 2010). Cytokines such as TNFα and IL6 impair synaptic plasticity and neurogenesis, partly through disruption of BDNF signaling (Jimenez et al. 2018; Miranda et al. 2019; Golia et al. 2019). Consistent with this, increased hippocampal inflammation and oxidative damage have been linked to anxiety‐like behavior in rodent models (Rammal et al. 2008; Fedoce et al. 2018; de Oliveira et al. 2007).

The postweaning period is marked by rapid brain development, including synaptic remodeling and maturation of neuroendocrine systems (Semple et al. 2013; Tau and Peterson 2010). During this stage, the brain is more sensitive to environmental and metabolic challenges (Gluckman et al. 2008; Barker 2007), thus, nutritional stressors in early development can produce more pronounced and persistent consequences (Spencer 2012). While OD withdrawal has been studied in adult animals (Casagrande et al. 2023; Cottone et al. 2008), much less is known about its effects during this developmental window, which are likely more affected by short‐term negative responses and could impact adherence to healthier diets (Birch and Fisher 1998).

In this study, we examined the short‐term effects of OD withdrawal in postweaning male Wistar rats. Animals were exposed to an OD for only 2 weeks, followed by a brief 48‐h withdrawal period. We hypothesized that even brief OD exposure, followed by withdrawal, would disrupt metabolic homeostasis and increase anxiety‐like behavior. This design allows the characterization of a short dietary transition during postweaning development, in which abrupt withdrawal after a brief obesogenic exposure may be associated with concurrent metabolic, inflammatory, oxidative, and behavioral alterations.

## 2 Methods

### 2.1 Ethics and Sample Size Calculations

The ethics committee of the Federal University of Sao Paulo (n° 9512270320) approved all procedures. National and international ethical principles and guidelines for animal research were followed. We calculated the sample size with G*Power software (version 3.1.9.7) considering a high effect size (*f* = 1 or *r* ^2^ = 0.50), with power (1 − *β*) at 80%, and alpha at 5% as the convention. The calculation returned a total of 15 animals. The effect size used for the calculations was extracted from previous data and considering the primary outcome “anxiety‐like behavior” on the elevated plus maze (EPM) (Casagrande et al. 2023) and hippocampal inflammation (Casagrande et al. 2022b).

### 2.2 Study Design

Postweaning male Wistar rats (3 weeks old) were used in this study. The animals underwent adaptation to the vivarium for 7 days. At 4 weeks of age (28 days), the rats were assigned to the treatment groups and received the control or OD and water for 14 days ad libitum (Table 1). The OD, a high‐sugar/high‐fat diet (HSHF), was adapted from our previous HSHF diet model (Casagrande et al. 2020) to meet the AIN‐93 protein recommendations for the growth period (Reeves et al. 1993). The HSHF diet was prepared by adding sweetened condensed milk (Nestlé) and lard (Sadia, São Paulo, Brazil) to ground control chow. To meet nutritional recommendations (Reeves et al. 1993), the formulation was adjusted with casein (Labsynth, Diadema, Brazil), soybean oil (Bunge, Gaspar, Brazil), and a vitamin and mineral mix (RHOSTER, Araçoiaba da Serra, Brazil). Additionally, to reflect the excess sodium intake observed globally, approximately double the recommended levels (Afshin et al. 2019; WHO 2012), sodium chloride was added to the diet (11.6 g/kg, corresponding to 4.5 g sodium) to achieve a twofold increase in sodium content in the final formulation. This amount was calculated based on the sodium content of the control diet (2700 mg/kg). While excess fat and sugar intake are the most studied components of ODs, high sodium intake is also a major contributor to diet‐related disease, largely driven by processed foods (WHO 2012).

**TABLE 1:** Diet composition.

Components (g/100 g)	High‐sugar/high‐fat diet	Control diet
Control diet	285.48	1000.00
Sweetened condensed milk	386.57	0.00
Lard	79.06	0.00
Casein	79.06	0.00
Sucrose	114.19	0.00
Soybean oil	10.76	0.00
Mineral mix (AIN‐93)	25.43	0.00
Vitamin mix (AIN‐93)	8.14	0.00
Butylhydroxytoluene	0.11	0.00
Sodium chloride	11.62	0.00
Sodium	5.27	2.70
**Nutrients (g/1000 g)**		
Carbohydrates	493.32	583.30
Sucrose	326.80	0
Fiber	72.43	253.70
Protein	138.93	209.70
Lipids	133.42	44.40
Added saturated fat	50.16	
**Energy (%)**		
Carbohydrates	48.94	51.56
Sucrose	38.00	0
Protein	16.15	32.81
Lipids	34.91	15.63
Saturated fat	13.12	0
**Energy (kcal/1000 g)**	3440.08	2556.80

Group allocation was performed using stratified randomization based on absolute body mass and its variation during the adaptation period. The animals were assigned to either the control diet group (*n* = 5) or the HSHF diet group (*n* = 10). The control group (Ct) was fed the control diet for 14 days, while the HSHF group was divided into a no‐withdrawal subgroup (NWt, *n* = 5), which was fed the HSHF diet for 14 days, and a withdrawal subgroup (Wt, *n* = 5), which was fed the HSHF diet for 14 days followed by the control diet for 2 days. As a result, the Wt remained under experimental conditions for an additional 48 h compared to the other groups, which represents a potential time‐related confounder. The animals were group‐housed (five animals/cage).

Food intake was measured every 2–3 days by calculating the difference between the amount of food provided and the sum of the remaining food and any food scattered in the bedding. It was expressed as grams consumed per animal per 24 h per 100 g of body mass. Energy intake was calculated according to food intake and diet composition (Table 1) and is reported as kcal consumed per animal per 24 h per 100 g of body mass. Body mass was evaluated at the beginning, each week, and at the end of the protocol. Also, we measured it every day of the 2nd week and during the 2 days of withdrawal. The percentage of body mass gained was calculated at each point accordingly.

At the end of the protocol behavioral tests were conducted. After a fasting period of 6–8 h, the animals were anesthetized with isoflurane (1.25 mL/L) in a sealed transparent plastic box and euthanized by decapitation after confirming complete anesthesia. Trunk blood, hypothalamus, hippocampus, liver, and visceral adipose tissues—mesenteric, epididymal, and retroperitoneal—were collected. The tissue masses were verified, and they were reported relative to body mass. In all analysis, the investigator was blinded to the treatment conditions.

### 2.3 Behavioral Testing

The animals were fasted for 1 h before testing while they adapted to the room. The tests began at 8:00 h, they were conducted in the same conditions across the whole experiment. The room had dimmed lights and controlled noise. The apparatus was cleaned with 70% and 5% alcohol and was left to dry after each run.

The open field (OF) and the EPM were used to determine anxiety‐like behavior. The OF consisted of a 60 cm circular arena subdivided into a central zone (30 cm diameter) surrounded by a peripheral zone (15 cm). Each animal was placed individually in the center of the apparatus and recorded for 5 min. Total lines crossed, time in the center, and lines crossed in the peripheral zone were evaluated with ANY‐maze (v7.4, Stoelting Co, USA). The EPM was set at 50 cm above the floor. The apparatus consisted of four intercalated arms—two open and two enclosed—each measuring 60  × 15 cm. The center of the apparatus was a square of complementary dimensions (15 × 15 cm). Each one of the animals was placed in the center facing the same open arm (OA) and it was recorded for 5 min with free access to the entire maze. Entries, distance, time, average speed, and immobility time in each section of the maze were evaluated with ANY‐maze (v7.4, Stoelting Co, USA).

To characterize the anxiety‐like behavior, the time in the center of the OF, and the parameters of the EPM, the percentage of entries, time, and distance on the OA (Pellow et al. 1985) in relation to the total number of entries, time, and distance on the whole apparatus, OA, enclosed arms (EA), and center (e.g., entries on the OA/sum of entries in the OA, EA, and center) were evaluated. Additionally, we evaluated risk assessment via protected and unprotected head dips, which was done manually by two independent blinded researchers. Then the principal component analysis (PCA) approach was used for further data interpretation to unify measures from both tests in a single component.

### 2.4 Serum Parameters

Trunk blood collected during the euthanasia after 6–8 h fasting was separated into serum (10 min centrifugation at 5000 × *g* at 4°C). Serum glucose (#133, Labtest, Brazil), insulin (80‐INSMR‐CH01, STELLUX, ALPCO, US), triacylglycerol (TAG) (#87, Labtest, Brazil), total cholesterol (#76, Labtest, Brazil), and HDL‐cholesterol (#13, Labtest, Brazil) were determined with colorimetric kits following the manufacturer's instructions. HOMA‐IR index and n‐HDL/HDL cholesterol ratio were calculated.

### 2.5 Hepatic Glycogen and Lipids

Hepatic glycogen levels were determined by adapting the micromethod proposed by Balmain et al. (1956), as previously standardized by our group (Casagrande et al. 2019). Hepatic lipids were extracted following the Folch et al. (1957) method. From those lipids, we quantified TAG, cholesterol, and HDL‐cholesterol using the above‐reported colorimetric kits (Labtest, Brazil).

### 2.6 Inflammation

Inflammatory cytokines were assessed in the hypothalamus, liver, hippocampus, serum, and mesenteric fat. Epididymal and retroperitoneal fat deposits were not evaluated since we found no important cytokine alterations in these tissues in the previous work (Casagrande et al. 2020). The serum was prepared for the analysis as previously described (Casagrande et al. 2022b). Tissues were homogenized in ice‐cold lysis buffer (100 mM Tris‐HCl, 10 mM sodium pyrophosphate, 10 mM sodium orthovanadate, 2 mM phenylmethylsulfonyl fluoride, 0.04% bovine lung aprotinin, 10 mM EDTA, 100 mM sodium fluoride, 1% Triton) and centrifuged at 20,000 × *g* for 40 min at 4°C. The supernatant was transferred to another microtube, and protein content was measured with Bradford reagent (Bradford 1976). The concentrations of IL6 (#DY506, R&D Systems, USA), IL10 (#DY522, R&D Systems, USA), IL1β (#DY501, R&D Systems, USA), and TNFα (#DY510, R&D Systems, USA) were evaluated in the supernatant following the manufacturer's instructions.

### 2.7 Oxidative Status

Hippocampi samples were homogenized in 0.2 M PBS (pH 7.4) and centrifuged at 4°C for 15 min at 5000 × *g*. The supernatant was used to estimate the activity of catalase (CAT), superoxide dismutase (SOD), and malondialdehyde (MDA) concentrations, the pellet was used to estimate the concentration of carbonylated proteins (CBP).

CAT activity was estimated as described by Góth (1991), which is based on hydrogen peroxide neutralization, the reaction was carried out for 3 min and interrupted by the addition of ammonium molybdate. The calculations were carried out to estimate the activity of CAT as hydrogen peroxide units consumed per minute, and it was adjusted by the protein content of the sample (Góth 1991).

SOD content was estimated by the autoxidation of pyrogallol and continued superoxide‐dependent reduction of 3‐(4,5‐dimethyl‐thiazol‐2‐yl) 2,5‐diphenyl tetrazolium bromide (MTT) to formazan. This reaction was incubated for 15 min at 37°C and was interrupted by the addition of dimethyl sulfoxide (DMSO). The results are shown as SOD units per mg of protein (Madesh and Balasubramanian 1998).

MDA concentration was estimated by the thiobarbituric acid reactive substance (TBARS) assay. Samples were incubated in a water bath at 90° for 40 min; we performed an alcoholic extraction with *n*‐butanol, and its phase was recovered by centrifugation for 10 min at 5000 × *g*. The results are shown as µM/mg protein (Schmedes and Hølmer 1989; Oakes and Van Der Kraak 2003).

Protein carbonylation was estimated by derivatization with 2,4‐dinitrophenylhydrazine (DNPH) in a 2 M HCl solution in the dark. The proteins were precipitated with a 10% trichloroacetic acid (TCA) solution and washed twice with equal parts ethyl acetate and ethanol solution. Between the washes, the samples were centrifuged (10 min, 4°C, 10,000 × *g*), and the supernatant was removed. In the final step, a 6% SDS solution was added, the sample was homogenized and centrifuged once again (10 min, 23°C, 10,000 × *g*), and the supernatant was used for the readings. A blank was run without the DNPH addition alongside each sample. After the calculations, the result is expressed in nmol/mL (Colombo et al. 2016).

### 2.8 Statistical Analysis

Data were represented as mean ± standard deviation, which are presented as median with interquartile range, with individual data points overlaid in graphical representations. For all analysis, jamovi software was used (version 2.6.2) alongside GAMLj3 module (3.4.2) (Galluci 2020). Due to the small sample size, no outlier exclusion procedures were applied; outliers were identified and winsorization was employed to preserve robustness and stabilize variance (Tukey 1962). We used generalized linear models (GZLM) and generalized estimated equations (GEE) to analyze single and multiple observation data, respectively. Distribution was selected based on data type and AIC. Holm's stepwise correction was used in all cases. To verify if the food and calorie intake was different upon withdrawal in the Wt group, the *Z*‐test was performed. Pearson's correlations were used to determine relationship between variables. Fisher *r*‐to‐*z* transformation was also employed to determine differences between observed correlations. The PCA was used to combine EPM variables to determine anxiety‐like behavior. Statistical significance was set at 5% as convention and the effect sizes are reported. For GZLM and GEE, *χ* ^2^ values, *p*‐value, residual degrees of freedom (rDf), and *r* ^2^ were reported; for between‐group comparisons, *p*‐value and Hedges’ *g* were reported. The sample size calculations were performed considering a power level (1 – *β* error) of 80%. To attend the matter of the sample size and concerns about the validity of the findings, it is expected that the *r* ^2^ is greater than 0.5 for GZLMs (single observation) and *r* ^2^ greater than 0.066 for GEEs (repeated observations), considering the required Cohen's *f* (1 and 0.266).

## 3 Results

### 3.1 Food Intake, Body Mass, and Adiposity

**FIGURE 1:**
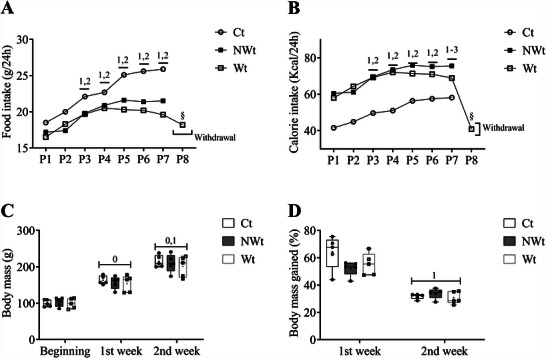
Food and calorie intake, body mass, and percentage body mass gained over time. (A) Food intake consumed (g) per 24 h per animal, the intake was measured every 2–3 days (*n* = 1 cage/group); P8 is withdrawal period. (B) Calorie intake consumed (kcal) per 24 h per animal, the intake was measured every 2–3 days (*n* = 1 cage/group); P8 is withdrawal period. (C) Body mass (g) per group each week (*n* = 5/group). (D) Body mass gained (%) per group each week (*n* = 5/group). (A, B) Numbers 1–3 indicate *p* < 0.05 compared to respective period (P) (1 to P1, 2 to P2); § indicates *p* < 0.05 for one‐tailed *Z*‐test compared to Wt intake. (C, D) Numbers 0 and 1 indicate *p* < 0.05 compared to the beginning (0) and 1st week (1).

Food and calorie intake are displayed in Figure 1A,B. Because animals were housed in a single cage per group, we could not analyze the group‐by‐time interaction; however, it is possible to determine the effects of group and time separately.

Food (*χ* ^2^ = 9.67, *p* < 0.001, rDf = 6, *r* ^2^ = 0.92) and calorie (*χ* ^2^ = 6.93, *p* = 0.002, rDf = 6, *r* ^2^ = 0.39) intake increased with time. Post‐hoc comparisons are indicated in Figure 1A,B. The HSHF‐fed groups consumed less chow than the Ct group (Ct vs. NWt *p* < 0.001, *g* = 2.57, Ct vs. Wt *p* < 0.001, *g* = 3.43). Conversely, they consumed more calories than Ct (Ct vs. NWt *p* < 0.001, *g* = −7.55, Ct vs. Wt *p* < 0.001, *g* = −6.52). At Period 8, the withdrawal for Wt, the food intake dropped in both kcal (two‐tail *Z*‐test *p* < 0.001) and grams (two‐tail *Z*‐test *p* = 0.042).

Initial and final body masses were similar among groups (Table 2 and Table S1). Although, body mass increased in all groups in the 1st and 2nd week compared to the previous (*χ* ^2^ = 506.90, *p* < 0.001, rDf = 24, *r* ^2^ = 0.84; beginning vs. 1st *p* < 0.001, *g* = −3.33; beginning vs. 2nd *p* < 0.001, *g* = −6.39; 1st vs. 2nd *p* < 0.001, *g* = 2.98) (Figure 1C). However, the percentage of gain decreased from the 1st to the 2nd week (*χ* ^2^ = 129.76, *p* < 0.001, rDf = 12, *r* ^2^ = 0.84; 1st vs. 2nd *p* < 0.001, *g* = 2.99). There were no differences across groups in either body mass (*χ* ^2^ = 0.39, *p* = 0.682, rDf = 12) or body mass gain (*χ* ^2^ = 1.42, *p* = 0.278, rDf = 12) progressions (Figure 1D).

**TABLE 2:** Body mass, adiposity, serum parameters, and metabolic parameters.

Parameter	Ct (mean ± SD)	NWt (mean ± SD)	Wt (mean ± SD)
**Initial body mass (g)**	99.80 ± 9.34	103.00 ± 12.49	99.20 ± 13.88
**Final body mass (g)**	215.00 ± 16.43	209.60 ± 24.76	211.80 ± 27.86
**Body mass gained (%)**	116.10 ± 14.61	103.89 ± 12.29	114.26 ± 17.59
**Adiposity**			
**Visceral fat (g/100 g)**	1.24 ± 0.13	**2.61 ± 0.20** ^a^	**2.39 ± 0.45** ^a^
**Mesenteric fat (g/100 g)**	0.39 ± 0.06	**0.63 ± 0.08** ^a^	**0.66 ± 0.15** ^a^
**Epididymal fat (g/100 g)**	0.47 ± 0.06	**0.97 ± 0.05** ^a^	**0.77 ± 0.11** ^a^, ^b^
**Retroperitoneal fat (g/100 g)**	0.38 ± 0.11	**1.02 ± 0.14** ^a^	**0.96 ± 0.23** ^a^
**Serum**			
**Glucose**	115.95 ± 5.95	117.65 ± 5.97	**104.9 ± 1.75** ^a^, ^b^
**Insulin**	0.25 ± 0.04	0.28 ± 0.15	**0.56 ± 0.04** ^a^, ^b^
**HOMA‐IR**	12.19 ± 1.55	14.25 ± 8.02	**25.81 ± 0.9** ^a^, ^b^
**Corticosterone (ng/mL)**	22.68 ± 9.26	**12.51 ± 4.94** ^a^	**69.69 ± 27.56** ^a^, ^b^
**Triacylglycerol (mg/dL)**	90.79 ± 2.28	**157.02 ± 8.28** ^a^	**46.32 ± 14.18** ^a^, ^b^
**Cholesterol (mg/dL)**	233.15 ± 61.56	236.31 ± 104.19	314.67 ± 62.5
**HDL cholesterol (mg/dL)**	44.34 ± 4.90	53.90 ± 10.36	**68.14 ± 11.20** ^a^
**n‐HDL cholesterol (mg/dL)**	214.53 ± 21.95	182.41 ± 100.74	246.53 ± 57.44
**Liver**			
**Glycogen (mg/g)**	1.75 ± 0.49	**8.40 ± 2.56** ^a^	**3.30 ± 0.88** ^a^, ^b^
**Triacylglycerol (mg/100 g)**	1.23 ± 0.02	**1.43 ± 0.16** ^a^	**1.21 ± 0.06** ^b^
**Cholesterol (mg/100 g)**	0.74 ± 0.08	0.70 ± 0.02	0.74 ± 0.07
**HDL cholesterol (mg/100 g)**	0.41 ± 0.01	0.39 ± 0.01	0.39 ± 0.02
**n‐HDL cholesterol (mg/100 g)**	0.33 ± 0.08	0.31 ± 0.01	0.35 ± 0.06

^a^ *p* < 0.05 compared to Ct.

^b^ *p* < 0.05 compared to NWt.

Overall body mass gained was also similar among groups, yet visceral adiposity (g/100 g) was different (Table 2). It was higher in HSHF‐fed groups compared to Ct (Ct vs. NWt *p* < 0.001, *g* = −3.60; Ct vs. Wt *p* < 0.001, *g* = −3.24). Mesenteric, epididymal, and retroperitoneal fat deposits were increased in NWt and Wt groups compared to Ct (mesenteric Ct vs. NWt *p* = 0.008, *g* = −2.72; Ct vs. Wt *p* = 0.004, *g* = −1.92; epididymal Ct vs. NWt *p* < 0.001, *g* = −8.86; Ct vs. Wt *p* < 0.001, *g* = −2.65; retroperitoneal Ct vs. NWt *p* < 0.001, *g* = −4.18; Ct vs. Wt *p* < 0.001, *g* = −2.58) (Table 2 and Table S1). Wt group presented intermediate values for epididymal fat, lower than NWt (*p* = 0.002, *g* = 1.86).

### 3.2 Serum and Hepatic Parameters

Serum glucose was lower in the Wt group compared to Ct and NWt (Ct vs. Wt *p* = 0.001, *g* = 2.10; NWt vs. Wt *p* < 0.001, *g* = 2.40). Alongside, Wt had higher insulin (Ct vs. Wt *p* = 0.002, *g* = −2.08; NWt vs. Wt *p* = 0.006, *g* = −2.31) and HOMA‐IR index (Ct vs. Wt *p* = 0.002, *g* = −8.71; NWt vs. Wt *p* = 0.004, *g* = −1.64) (Table 2 and Table S1).

NWt group had higher serum TAG compared to the other groups (Ct vs. NWt *p* < 0.001, *g* = −8.82 NWt vs. Wt *p* < 0.001, *g* = 7.71). Wt presented lower TAG than the Ct group (Ct vs. Wt *p* < 0.001, *g* = 3.54). HDL cholesterol was higher in Wt compared to Ct (Ct vs. Wt *p* = 0.005, *g* = −2.22). No alterations were seen in total or n‐HDL cholesterol (Table 2 and Table S1).

Hepatic glycogen was higher in the NWt group compared to the other groups (Ct vs. NWt *p* < 0.001, *g* = −2.91; NWt vs. Wt *p* < 0.001, *g* = 2.15). The Wt also presented higher glycogen than the Ct group (Ct vs. Wt *p* = 0.003, *g* = −1.76). Hepatic TAG was also higher in NWt compared to the other groups (Ct vs. NWt *p* = 0.015, *g* = −1.42; NWt vs. Wt *p* = 0.010, *g* = 1.52) (Table 2 and Table S1).

### 3.3 Inflammation

**FIGURE 2:**
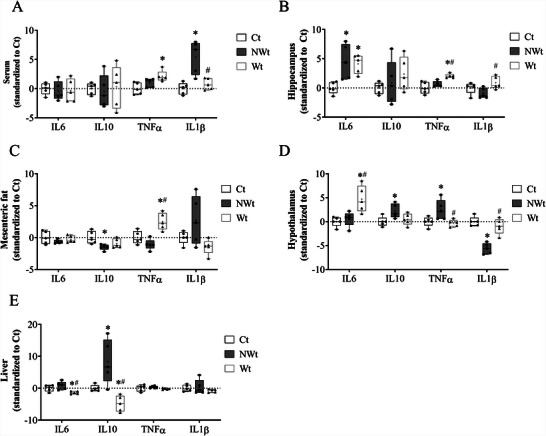
Graphical representation of standardized values of cytokines. (A) Serum, (B) hippocampus, (C) mesenteric fat, (D) hypothalamus, and (E) liver. * indicates *p* < 0.05 compared to Ct; ^#^ indicates *p* < 0.05 compared to NWt. Cytokine values are shown as *Z*‐scores to facilitate comparison across tissues and cytokines with different concentration ranges; absolute values are reported in Table S3.

In the serum, IL6 and IL10 were not different among groups. There was an increase in serum TNFα in the Wt group compared to Ct (Ct vs. Wt *p* = 0.010, *g* = −1.73). Serum IL1β was higher in the NWt group compared to the other groups (Ct vs. NWt *p* = 0.003, *g* = −2.04; NWt vs. Wt *p* = 0.008, *g* = 1.76) (Figure 2A and Table S2).

In the hippocampus, NWt and Wt presented higher IL6 levels compared to Ct (Ct vs. NWt *p* = 0.007, *g* = −1.65; Ct vs. Wt *p* = 0.007, *g* = −2.49). TNFα was higher in the Wt group compared to the other groups (Ct vs. Wt *p* = 0.004, *g* = −2.18; NWt vs. Wt *p* = 0.037, g = −2.55). Wt showed an increase in IL1β compared to NWt (NWt vs. Wt *p* = 0.040, *g* = −1.49) (Figure 2B and Table S2).

In mesenteric fat, IL10 was lower in NWt compared to Ct (*p* = 0.023, *g* = 1.56). TNFα was higher in the Wt group compared to the other groups (Ct vs. Wt *p* = 0.007, *g* = −1.72; NWt vs. Wt *p* < 0.001, *g* = −2.61) (Table 3) (Figure 2C and Table S2).

**TABLE 3:** Hippocampal oxidative stress.

Parameter	Ct (mean ± SD)	NWt (mean ± SD)	Wt (mean ± SD)
**SOD (units/mg of protein)**	209.11 ± 12.99	217.69 ± 31.69	**334.50 ± 52.86** ^a^, ^b^
**CAT (H_2_O_2_ units consumed/min/mg of protein)**	0.34 ± 0.13	0.35 ± 0.09	0.35 ± 0.03
**MDA (µmol/mg of protein)**	0.17 ± 0.02	0.17 ± 0.01	**0.23 ± 0.05** ^a^, ^b^
**CBP (mmol/mL)**	1.60 ± 0.43	**0.65 ± 0.21** ^a^	**0.51 ± 0.14** ^a^

Abbreviations: CAT, catalase; CPB, carbonylated protein; MDA, malondialdehyde; SOD, superoxide dismutases.

^a^ *p* < 0.05 compared to Ct.

^b^ *p* < 0.05 compared to NWt.

In the hypothalamus, IL6 was higher in Wt compared to Ct and NWt (Ct vs. Wt *p* = 0.006, *g* = −1.48; NWt vs. Wt *p* = 0.010, *g* = −1.81). IL10 was higher in NWt compared to Ct (Ct vs. NWt *p* = 0.049, *g* = −1.42). TNFα was also increased in NWt group (Ct vs. NWt *p* = 0.032, *g* = −1.21; NWt vs. Wt *p* = 0.022, *g* = 1.44). Conversely, IL1β presented an opposite response, NWt showed lower concentration than Ct and Wt (Ct vs. NWt *p* < 0.001, *g* = −4.42; NWt vs. Wt *p* < 0.001, *g* = −2.69) (Figure 2D and Table S2).

In the liver, Wt presented a decrease in IL6 and IL10 content compared to Ct (IL6 *p* = 0.031, *g* = 1.48; IL10, *p* = 0.047, *g* = 2.07) and NWt group (IL6, *p* = 0.005, *g* = 1.94; IL10, *p* < 0.001, *g* = 2.04). NWt showed higher concentrations than Ct group (*p* = 0.016, *g* = −1.36) (Figure 2E and Table S2).

### 3.4 Hippocampal Oxidative Status

Hippocampal SOD was higher in the Wt group compared to Ct and NWt (Ct vs. Wt *p* < 0.001, *g* = −2.65; NWt vs. Wt *p* < 0.001, *g* = −2.24) and the same pattern was seen for MDA (Ct vs. Wt *p* = 0.022, *g* = −1.23; NWt vs. Wt *p* = 0.013, *g* = −1.50). CBP was lower in NWt and Wt groups compared to Ct (Ct vs. NWt *p* = 0.001, *g* = 2.29; Ct vs. Wt *p* < 0.001, *g* = 2.82) (Table 3 and Table S2).

### 3.5 Behavioral Testing

**FIGURE 3:**
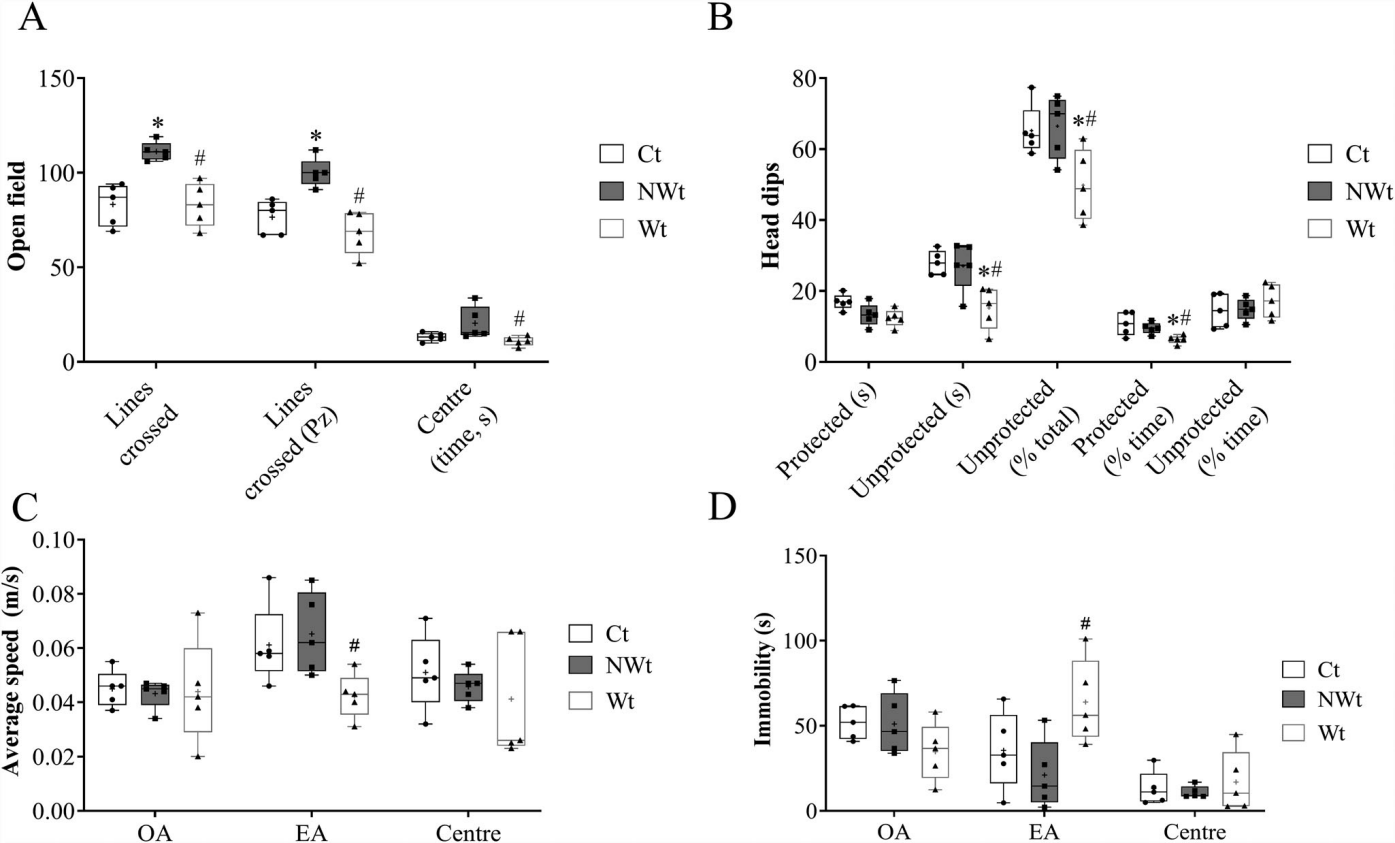
Graphical representation of behavioral parameters. (A) Open field lines crossed and time in the center; (B) risk assessment in the elevated plus maze as protected and unprotected head dips; (C) average speed in the sections of the elevated plus maze; and (D) immobility time in the sections of the elevated plus maze. * indicates *p* < 0.05 compared to Ct; ^#^ indicates *p* < 0.05 compared to NWt. EA, enclosed arms; OA, open arms.

Total lines crossed on the OF was higher in the NWt (*p* = 0.006, *g* = −2.93) group but decreased upon withdrawal (*p* = 0.006, *g* = 2.86). The same effect was observed with lines crossed on the peripheral zone (Ct vs. NWt *p* = 0.012, *g* = −2.53; NWt vs. Wt *p* = 0.002, *g* = 2.99). Time in the center of the OF was lower on the Wt group compared to NWt (*p* = 0.027, *g* = −1.36) (Figure 3A and Table S4). No differences were seen in zone crossing and entries in both regions (data not shown).

**FIGURE 4:**
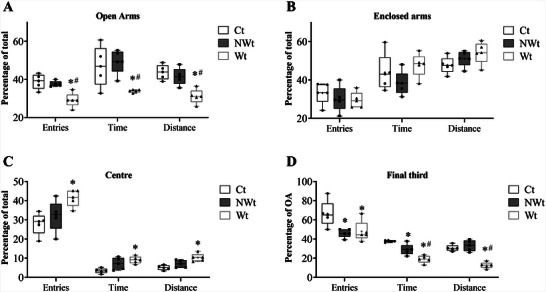
Graphical representation of elevated plus maze. (A) entries (% total), time (% total), and distance (% total) on the open arms; (B) entries (% total), time (% total), and distance (% total) on the enclosed arms; (C) entries (% total), time (% total), and distance (% total) on the center; (D) entries (% open arms), time (% open arms), and distance (% open arms) on the final third of the open arms. * indicates *p* < 0.05 compared to Ct; ^#^ indicates *p* < 0.05 compared to NWt.

Total distance in the EPM was lower in the Wt group compared to both Ct and NWt (average [± SD], Ct 2.10 ± 0.17; NWt 2.06 ± 0.17; Wt 1.22 ± 0.25) (*χ* ^2^ = 62.09, *p* < 0.001, rDf = 2, *r* ^2^ = 0.84; Ct vs. Wt *p* < 0.001, *g* = 3.56; NWt vs. Wt *p* < 0.001, *g* = 3.53). In the OA of the EPM, the Wt group presented lower percentages of entries (Ct vs. Wt *p* = 0.001, *g* = 2.04; NWt vs. Wt *p* = 0.003, *g* = 2.41), time (Ct vs. Wt *p* = 0.011, *g* = 1.41; NWt vs. Wt *p* = 0.007, *g* = 2.80), and distance (Ct vs. Wt *p* = 0.001, *g* = 2.68; NWt vs. Wt *p* = 0.002, *g* = 1.64) than the Ct and NWt groups (Figure 4A and Table S4). No differences were seen in the EA (Figure 4B and Table S4). To determine whether reduced exploration reflected anxiety‐like behavior or locomotor impairment, we analyzed average speed and immobility across EPM. No differences were observed in either parameter in the OA or center. In the EA, the Wt group exhibited lower average speed and increased immobility; however, these effects were associated with low *r* ^2^ and insufficient statistical power (Figure 3C,D and Table S4).

In the center (Ce), the NWt group showed higher percentages of time (*p* = 0.023, *g* = −1.26) than the Ct group. While the Wt group presented higher percentages of entries (*p* = 0.029, *g* = −2.16), time (*p* = 0.009, *g* = −2.92), and distance (*p* = 0.004, *g* = −2.48) compared to the Ct group (Figure 4C and Table S4).

In the final third of the OA, the NWt and Wt groups showed lower percentage of entries (Ct vs. NWt *p* = 0.032, *g* = 1.63; Ct vs. Wt *p* = 0.038, *g* = −1.21) and time than Ct group (Ct vs. NWt *p* = 0.030, *g* = −1.72; Ct vs. Wt *p* < 0.001, *g* = −5.23). Whereas Wt groups showed a further decrease compared to NWt in the percentage of time (*p* = 0.004, *g* = 1.64). Wt groups also had lower percentage of distance traveled in the final third compared to both Ct and NWt (Ct vs. Wt *p* < 0.001, *g* = 4.59; NWt vs. Wt *p* < 0.001, *g* = 3.64) (Figure 4D and Table S3).

The percentage of time in the OA was negatively correlated with the percentage of time spent in the EA (*r* = −0.786, *r* ^2^ = 0.61, *p* < 0.001), while the percentage of distance on the OA was negatively correlated with the percentage of distance on the EA (*r* = −0.792, *r* ^2^ = 0.627, *p* < 0.001). The correlations were adjusted for group conditions.

Risk assessment was also different among groups. Wt group showed lower head dips in the OA, as unprotected head dips, compared to both Ct (*p* = 0.011, *g* = 2.38) and NWt (*p* = 0.012, *g* = 1.66). While there was a trend (*p* = 0.053, *g* = 1.78) toward lower protected head dips compared to Ct group. Wt group also showed a lower percentage of unprotected head dips, as a percentage of total head dips, compared to both Ct (*p* = 0.033, *g* = 1.60) and NWt (*p* = 0.033, *g* = 1.66). As the time in each section of the EPM was different among groups, we calculated the percentage of time on head dips in the protected and unprotected sections. With this approach, Wt group presented lower protected head dips considering time under protected conditions—at the center or EA—compared to both Ct (*p* = 0.012, *g* = 2.07) and NWt (*p* = 0.033, *g* = 1.65), but no differences were seen for unprotected head dips in the same conditions (Figure 3B and Table S4).

For the anxiety‐like behavior determined via PCA, named as ANX, its component comprised the following variables and had the following component loadings: entries, distance, and time in the OA (entries: −0.79; distance: −0.94; time: −0.87), distance and time in the EA (distance: 0.77; time: 0.81) related to totals on the apparatus; protected (−0.57) and unprotected head dips (−0.94); and time in the center of the OF (−0.45). The greater the score, the greater the anxiety‐like behavior. ANX (average [± SD], Ct −0.59 ± 0.74; NWt −0.49 ± 0.62; Wt 1.08 ± 0.62) was higher in the Wt group compared to both Ct (*p* = 0.005, *g* = −2.21) and NWt (*p* = 0.006, *g* = −2.29).

### 3.6 Correlations for Hypothalamic Cytokines and Energy Homeostasis

Several correlations were seen among hypothalamic cytokines and energy homeostasis parameters evaluated. For all groups, hypothalamic IL6 was correlated with insulin (*r* = 0.77, *p* < 0.001, 95% CI: 0.43–0.92), HOMA‐IR (*r* = 0.74, *p* = 0.002, 95% CI: 0.36–0.91), corticosterone (*r* = 0.65, *p* = 0.009, 95% CI: 0.20–0.87), serum glucose (*r* = −0.53, *p* = 0.043, 95% CI: −0.02 to −0.82), and serum TAG (*r* = −0.54, *p* = 0.037, 95% CI: −0.04 to −0.82). As OD intake altered these parameters, we evaluated if the observed correlations differed when analyzing them separately. For the HSHF‐fed groups, the correlations were mostly maintained apart from corticosterone and glycemia (insulin *r* = 0.74, *p* = 0.014, 95% CI: 0.21–0.94; HOMA‐IR *r* = 0.69, *p* = 0.027, 95% CI: 0.11–0.92; corticosterone *r* = 0.61, *p* = 0.062; serum glucose *r* = −0.54, *p* = 0.107; serum TAG *r* = −0.69, *p* = 0.028, 95% CI: −0.92 to −0.10).

The HSHF‐fed groups also presented opposing correlations of these parameters with hypothalamic TNFα (insulin *r* = −0.82, *p* = 0.004, 95% CI: −0.96 to −0.39; HOMA‐IR *r* = −0.79, 95% CI: −0.95 to −0.39, *p* = 0.006; corticosterone *r* = −0.72, *p* = 0.018, 95% CI: −0.93 to −0.17; serum glucose *r* = 0.64, *p* = 0.044, 95% CI: 0.02–0.91; serum TAG *r* = 0.69, *p* = 0.027, 95% CI: 0.11–0.92). To confirm the significance of the opposing nature of the relationships, we applied the Fisher *r*‐to‐*z* transformation. All correlations were significantly different (insulin *z* = 3.94, *p* = 0.0001; HOMA‐IR *z* = 3.59, *p* = 0.0003; corticosterone *z* = 3.02, *p* = 0.0025; serum glucose *z* = −2.55, *p* = 0.0108; serum TAG *z* = −3.17, *p* = 0.0015).

While all groups are taken into account, only hypothalamic TNFα correlation with corticosterone and TAG are seen (insulin *r* = −0.50, *p* = 0.054; HOMA‐IR *r* = −0.49, *p* = 0.061; corticosterone *r* = −0.57, *p* = 0.027, 95% CI: −0.84 to −0.08; serum glucose *r* = 0.43, *p* = 0.107; serum TAG *r* = 0.65, *p* = 0.008, 95% CI: 0.21–0.87).

### 3.7 Correlations for Anxiety‐Like Behavior

The relevant correlations found are presented in Table 4. It is worth highlighting that parameters indicating a more anxious‐like phenotype were correlated with an inflammation hallmark, TNFα, in the serum, hippocampus, and mesenteric fat. Additionally, they were correlated with the oxidative status of the hippocampus, being directly associated with the increase in MDA and SOD. Likewise, it was directly correlated with serum corticosterone, a key marker of stress system activation.

**TABLE 4:** Correlations between inflammation, oxidative stress, and the open arms parameters.

	Serum Cort.	Serum TNFα	Hepatic IL6	Ht IL6	Hc TNF	M.FAT TNFα	Hc SOD	Hc MDA
Center (OF)								
Time (s)			0.696^*^			−0.514^*^		
Open arms (EPM)								
Entries (% total entries)	−0.801^*^	−0.623^*^		−0.699^*^	−0.515^*^	−0.765^*^	−0.727^*^	−0.526^*^
Time (% total time)	−0.685^*^		0.723^*^	−0.704^*^		−0.589^*^	−0.753^*^	
Distance (% total distance)	−0.675^*^		0.662^*^	−0.815^*^		−0.577^*^	−0.636^*^	
Final third of the OA (EPM)								
Time (% open arms)	−0.656^*^	−0.694^*^		−0.710^*^	−0.718^*^	−0.567^*^	−0.718^*^	−0.561^*^
Distance (% open arms)	−0.762^*^		0.812^*^	−0.794^*^	−0.591^*^	−0.691^*^	−0.834^*^	−0.661*
Unprotected head dips (EPM)	−0.735^*^	−0.558^*^	0.616^*^	−0.852^*^		−0.667^*^	−0.630^*^	
ANX (PCA)	0.704^*^		0.709^*^	0.839^*^		0.645^*^	0.743^*^	

Abbreviations: ANX, anxiety‐like behavior; Cort, corticosterone; Hc, hippocampal; Ht, hypothalamic; MDA, malondialdehyde; M.FAT, mesenteric fat; OF, open field; PCA, principal component analysis; SOD, superoxide dismutase.

^*^ *p* < 0.05.

## 4 Discussion

In this study, we evaluated the metabolic and behavioral effects of withdrawing from an OD after 2 weeks of intake in a postweaning model. In the present postweaning model, withdrawal was associated with rapid metabolic disruption and the emergence of anxiety‐like behavior within 48 h. These results indicate that metabolic and behavioral systems in early life can respond strongly to abrupt dietary change, even after brief exposure. Importantly, these effects did not occur in isolation. These outcomes occurred concurrently, suggesting that multiple metabolic, inflammatory, oxidative, and behavioral responses were engaged in parallel during abrupt dietary transition, rather than implying a single causal pathway. The temporal convergence of these outcomes suggests a coordinated physiological response to abrupt dietary transition rather than independent alterations across systems.

### 4.1 Metabolic and Inflammatory Effects of 2‐Week OD

Food intake (g) was higher in the Ct group, but calorie intake was higher in groups consuming the HSHF diet. Despite higher caloric intake over 2 weeks, body mass did not differ between groups. This likely reflects the rapid growth phase at this age (Iossa et al. 1999), and suggests changes in body composition, supported by higher visceral adiposity (Table 2).

Two weeks of HSHF intake caused dyslipidemia, increased hepatic glycogen, hepatic TAG deposition, and increased hypothalamic and serum inflammation. These findings partially contrast with previous studies in which 1‐ and 4‐week OD feeding did not consistently increase hepatic TAG (Chiba et al. 2016). In addition, while 4 weeks of OD intake elevated inflammation in the liver and mesenteric adipose tissue, the 2‐week protocol was insufficient to induce similar changes (Casagrande et al. 2020). Adolescent rats show greater hepatic lipogenic capacity, including sustained increases in fatty acid synthase and stearoyl‐CoA desaturase activity after short‐term fructose feeding (Mazzoli et al. 2021), but they may also show reduced baseline and stimulated pro‐inflammatory responses (Doremus‐Fitzwater et al. 2015). Younger animals may therefore be more sensitive to early metabolic alterations, including hepatic glycogen accumulation and TAG deposition (Casagrande et al. 2019).

The magnitude of changes after 2 weeks of OD intake indicates rapid metabolic disruption in early life, including a 110% increase in visceral adiposity, a 16% increase in hepatic lipid accumulation, and a 380% increase in hepatic glycogen. Although hepatic inflammation was not detected, the accumulation of visceral fat and hepatic glycogen likely reflects early adaptive responses to caloric excess. This supports the view that short‐term OD exposure can produce meaningful metabolic changes, even if inflammatory changes are not yet present. While some reports suggest inflammation precedes and may contribute to hepatic TAG accumulation (Casagrande et al. 2020; Heida et al. 2021), our pattern is consistent with the natural history of fatty liver (Fazel et al. 2016).

The absence of a direct anxiogenic effect after 14 days of OD intake is consistent with prior work. Vega‐Torres et al. (2020) reported that 11 days of a high‐fat diet during adolescence did not affect anxiety‐like behavior in the EPM, although findings across studies can be heterogeneous (Casagrande and Estadella 2020; Tsan et al. 2021), and are likely driven by differences in diet composition, such as fat and sugar content.

### 4.2 Acute Metabolic and Behavioral Shifts Upon Diet Withdrawal

Upon withdrawal, animals voluntarily decreased calorie intake, which may reflect lower preference for non‐palatable foods and/or the lower calorie density of the control diet (Casagrande and Estadella 2020; Martire et al. 2014). Visceral adiposity remained elevated compared to Ct, indicating that short‐term withdrawal did not reverse the adiposity increase induced by the HSHF diet.

After withdrawal, animals showed metabolic and behavioral changes consistent with a shift from an anabolic to a catabolic state. Compared with prior findings in older male Wistar rats exposed to 4 weeks of HSHF feeding (Casagrande et al. 2022b; Casagrande et al. 2020; Casagrande et al. 2022a), the 2‐day withdrawal after 2‐week OD intake showed a distinct metabolic response. This likely reflects differences in developmental stage, duration of exposure, and available energy stores, rather than a directly greater effect (Iossa et al. 1999; Santos et al. 2018; de Castro et al. 2013).

A briefer OD intake provides less opportunity for energy storage, and thus weaker “protection” to low energy availability states, which may be the cause for an amplified negative physiological impact to the OD removal. Consistently, the 48‐h withdrawal did not restore metabolic homeostasis and was associated with poor glucose and lipid handling, with a state of hypoglycemia, increased insulin and HOMA‐IR, reduced circulating TAG, and persistent visceral adiposity.

As expected, anxiety‐like behavior increased after OD withdrawal. In the OF, withdrawal animals differed from NWt, and in the EPM they showed reduced OA exploration and altered head dip measures compared with Ct and NWt. Effect sizes were large and comparable to prior work. The overall component (ANX) shows a consistent response. In the present study, the model effect size was *r* ^2^ = 0.63 (bootstrap 95% CI: 0.265–0.836), while a previous study reported *r* ^2^ = 0.47. Between‐group comparisons also showed Hedges’ *g* values within the reported range. Compared to control groups, we observed *g* = −2.21 (95% CI: −3.38 to −0.54), while a previous study reported *g* = −1.50; compared to animals still on OD, we observed *g* = −2.29 (95% CI: −3.91 to −0.59), while a previous study reported −2.19 (Casagrande et al. 2023).

In adult mice, the trajectory appears different. Garcia‐Serrano et al. (2022) reported that long‐term OD exposure induces metabolic and behavioral impairments that are largely reversed after prolonged diet normalization. This suggests that in mature systems, diet‐induced alterations can be reversible with sufficient recovery time. Our findings are not directly comparable due to differences in developmental stage and recovery duration, but together they indicate that response dynamics depend on age and timescale. The stronger response observed after short‐term exposure may be related to lower energy storage (e.g., visceral fat) compared to longer feeding protocols, which could increase sensitivity to abrupt reductions in caloric intake.

Reduced OA exploration can be confounded by locomotor changes, but this is unlikely here. Average speed and immobility time in the OA were unchanged, suggesting preserved motor capacity in the anxiogenic context. Modest changes in EA (reduced speed and increased immobility) had insufficient statistical power and may reflect increased passivity (e.g., coping or risk avoidance) rather than generalized hypoactivity. Neophobia is another potential confound, but prior work shows increased anxiety‐like behavior after sucrose withdrawal in paradigms that minimize neophobia (Kim et al. 2018). Similarly, Sharma et al. (2013) reported increased anxiety‐like behavior 1 day after high‐fat diet withdrawal, but not after removal of a low‐fat diet. Accordingly, the rodents were previously familiar with the control diet, which they were fed during acclimatization.

### 4.3 Neuroinflammation, Oxidative Stress, and Emergence of Anxiety‐Like Behavior

NWt animals showed higher serum IL1β, a systemic contributor to atherosclerosis (Libby 2017), but lower hypothalamic IL1β than Ct. In contrast, hypothalamic TNFα was elevated. Different hypothalamic inflammatory patterns are associated with distinct energy homeostasis profiles. Stimuli from saturated fatty acids and nutrient excess are linked to diet‐induced obesity, whereas stimuli derived from plasma cytokines/LPS are associated with sickness behavior and hypophagia (Thaler et al. 2010). TNFα can modulate insulin and leptin signaling and exert anorexigenic effects (Romanatto et al. 2007), while IL1β regulates cephalic insulin release (Wiedemann et al. 2022).

Following withdrawal, Wt animals showed a spike in serum TNFα compared to Ct and increased hypothalamic IL6 compared to both groups, consistent with prior findings (Casagrande et al. 2022a). Hypothalamic IL6 has been shown to increase fatty acid oxidation in skeletal muscle (Katashima et al. 2022), which may reflect a response to reduced energy availability. IL6 can also suppress feeding and improve peripheral glucose homeostasis (Timper et al. 2017), consistent with the observed reduction in intake and metabolic changes.

Hypothalamic IL6 correlated positively with serum insulin, insulin sensitivity, and corticosterone, and negatively with glycemia and serum TAG. These associations are consistent with IL6 being linked to coordinated central and peripheral responses during withdrawal, but they do not establish causality. The positive correlations may reflect an adaptive response to reduced energy availability, while the negative correlations may reflect compensatory changes in peripheral energy utilization, potentially involving IL6‐related increases in fatty acid oxidation (Katashima et al. 2022).

Within the HSHF‐fed groups, most correlations were sustained, and additional associations emerged: hypothalamic TNFα correlated negatively with insulin (*r* = −0.82, *p* = 0.004), HOMA‐IR (*r* = −0.80, *p* = 0.006), corticosterone (*r* = −0.72, *p* = 0.018), alongside positive correlations with glycemia (*r* = 0.64, *p* = 0.045) and serum TAG (*r* = 0.69, *p* = 0.027). These patterns highlight the complexity of cytokine interactions in the hypothalamus during withdrawal. While TNFα is often linked to inflammation and insulin resistance (Sethi and Hotamisligil 2021), the direction of associations here may reflect context‐dependent regulation during acute dietary transition. IL6 and TNFα patterns suggest potentially opposing roles in response to OD withdrawal. These correlations were observed primarily during the withdrawal condition and were not uniformly present during continued HSHF feeding.

The hippocampus was selected due to its established role in stress regulation and anxiety‐related behavior (Fanselow and Dong 2010; Jimenez et al. 2018; Jacobson and Sapolsky 1991), and its vulnerability to oxidative stress and inflammation (Salim 2017). After OD withdrawal, hippocampal pro‐inflammatory cytokines and oxidative stress markers were elevated compared to Ct and NWt. TNFα can disrupt glutamatergic signaling and reduce synaptic plasticity (Liu et al. 2017; Pickering et al. 2005), and elevated IL6 can exacerbate neuroimmune activation (Kummer et al. 2021). These mechanisms could contribute to altered hippocampal function relevant to anxiety‐like behavior, but this remains a hypothesis based on known biology rather than direct evidence in this study. The findings are consistent with literature linking neuroinflammation to reduced BDNF expression and impaired neurogenesis and synaptic connectivity (Golia et al. 2019; Dadkhah et al. 2024), processes implicated in anxiety disorders.

OD withdrawal also induced hippocampal oxidative stress, with elevated MDA and SOD compared to Ct and NWt. MDA reflects lipid peroxidation and oxidative damage to neuronal membranes, while increased SOD likely reflects a compensatory response to increased ROS. Hippocampal oxidative stress and inflammatory markers, as well as serum corticosterone correlated with anxiety‐like behaviors in the EPM (Table 4), supporting an association between these biological changes and the behavioral phenotype. Increased serum corticosterone is consistent with stress‐axis activation, which can worsen hippocampal oxidative stress and impair neuroplasticity. These results align with mechanisms implicated in anxiety‐related and neuropsychiatric conditions (Jones et al. 2022), but further work is needed to test causal pathways.

### 4.4 Implications for Dietary Transition and Intervention Strategies

Negative consequences of OD can manifest within 24 h (Enriquez et al. 2022) and persist as long as tested, although outcomes vary with sex, age, and diet composition and can change over time. Hence, short‐term withdrawal should not be interpreted as the beginning of the recovery phase. Instead, the present findings indicate that early withdrawal represents a distinct transitional state marked by physiological stress, which differs from both continued OD exposure and long‐term dietary normalization.

The 48‐h withdrawal protocol produced both expected and unexpected outcomes. While prior studies report metabolic and behavioral impacts of OD exposure, we did not expect such pronounced negative consequences after such short exposure. These findings suggest an early onset of mechanisms underlying the anxiogenic response to OD withdrawal, which indicates that prolonged exposure may not be necessary for addiction‐related behaviors to emerge in this early‐life stage.

At the same time, long‐term dietary improvements provide substantial benefits, highlighting the need for supporting strategies during early phases of dietary change. Pharmacotherapy and bariatric surgery can be effective in adults but are not primarily recommended for younger populations (No. 189 NICE Guideline 2023; Singhal et al. 2021). Therefore, developing and evaluating non‐pharmacological and behavioral interventions tailored to early life and to the early withdrawal period are needed.

## 5 Limitations

This study should be interpreted as characterizing short‐term withdrawal responses during early development rather than long‐term outcomes or permanent alterations. First, the model targets the early postweaning period, when neural and metabolic systems are still maturing. The responses likely reflect interactions between acute metabolic effects and developmental plasticity and may not generalize to fully mature organisms. Second, the withdrawal period was limited to 48 h to capture early‐phase responses; it does not address longer‐term recovery trajectories and is not directly comparable to studies using extended normalization periods. Third, animals were housed in a single cage per group, introducing potential cage‐level effects, including social hierarchy and shared microenvironment, which cannot be excluded post hoc. However, the consistency of outcomes across metabolic, inflammatory, and behavioral endpoints suggests the phenotype is unlikely to be driven by a single confounder. Fourth, despite being based on sample size calculations, the small group sizes (*n* = 5 per condition) limit generalization. Fifth, only male animals were included, limiting extrapolation across sexes, given known sex‐specific differences in response to ODs. Finally, the dietary model was adapted to approximate key features of ultra‐processed diets (high fat, sugar, and sodium). While this increases translational relevance, it reduces standardization relative to purified diets and may limit direct comparison with defined formulations.

## Author Contributions

**Breno Picin Casagrande**: conceptualization, data curation, formal analysis, funding acquisition, investigation, methodology, validation, writing – original draft, writing – review and editing. **Debora Estadella**: conceptualization, funding acquisition, resources, writing – review and editing. Luciana **Pellegrini Pisani**: funding acquisition, resources, writing – review and editing. **Vitória Rios Beserra**: formal analysis, methodology, writing – original draft. **Alessandra Mussi Ribeiro**: funding acquisition, resources, writing – review and editing.

## Funding

This work was supported by the “São Paulo Research Foundation” (FAPESP, #2021/02325‐0) and the “Coordination for the Improvement of Higher Education Personnel” (CAPES Brazil—Financial Code 001). L.P.P. is a beneficiary of the “National Council for Scientific and Technological Development” (CNPq) productivity fellowship. B.P.C. received a PhD scholarship from the São Paulo Research Foundation (FAPESP, #2019/22511‐3) during the development of this work and is currently supported by a FAPESP postdoctoral fellowship (FAPESP, #2025/08387‐9).

## Ethics Statement

The institutional ethics committee approved the present study, CEUA application n° 9512270320, certifiable at: http://ceua.sirpp.unifesp.br/.

## Conflicts of Interest

The authors declare no conflicts of interest.

## Supporting information




**Supplementary table 1**. Model information on single observation data parameters: body mass, adiposity, and metabolic.
**Supplementary table 2**. Model information on single observation data parameters: inflammation and oxidative stress.
**Supplementary table 3**. Cytokines absolute concentrations.
**Supplementary table 4**. Model information on single observation data parameters: anxiety‐like behaviour.
**Supplementary table 5**. 95% Confidence interval for correlations between inflammation, oxidative stress, and the open arms parameters.

## Data Availability

The data of the present work will be available at request to the corresponding author under reasonable conditions.
