# Supplementary material for: Rapid Metabolic and Behavioral Maladaptations Following Short‐Term Obesogenic Diet Withdrawal During Postweaning Development in Male Wistar Rats
Source: Brain Behav. 2026 Jul 9;16(7):e71492. doi: 10.1002/brb3.71492 (PMC13347161; doi:10.1002/brb3.71492)
Supplement: Supplementary file 1 — Supplementary table 1. Model information on single observation data parameters: body mass, adiposity, and metabolic. Supplementary table 2. Model information on single observation data parameters: inflammation and oxidative stress. Supplementary table 3. Cytokines absolute concentrations. Supplementary table 4. Model information on single observation data parameters: anxiety‐like behaviour. Supplementary table 5. 95% Confidence interval for correlations between inflammation, oxidative stress, and the open arms parameters. [file BRB3-16-e71492-s001.docx]

**Supplementary information**

**Rapid metabolic and behavioural maladaptations following short-term obesogenic diet withdrawal during post-weaning development in male Wistar rats.**

Breno Picin Casagrande^a^, Vitória Rios Beserra^a^, Alessandra Mussi Ribeiro^a^, Luciana Pellegrini Pisani^a^, Debora Estadella^a^.

^a^ Biosciences Department, Institute of Health and Society, Federal University of São Paulo, 1015-020, Santos, São Paulo, Brazil

ORCID / Email addresses:

0000-0001-9478-8262 / breno.casagrande@unifesp.br

0000-0002-2452-3258 / rios.vitoria@unifesp.br

0000-0002-7697-5766 / alessandra.ribeiro@unifesp.br

0000-0001-6579-6167 / pisani@unifesp.br

0000-0001-9853-3662 / estadella@unifesp.br*

*Corresponding author at Biosciences Department, Institute of Health and Society, Federal University of São Paulo, Campus Baixada Santista – UNIFESP/BS, Santos, 11015-020, SP, Brazil.

| **Supplementary table 1. Model information on single observation data parameters: body mass, adiposity, and metabolic.** | |
| --- | --- |
| Dependant variable | Model information |
| Initial body mass | χ² = 0.28, p = 0.866, rDf = 12, r² = 0.02, pw = 0.068 |
| Final body mass | χ² = 0.13, p = 0.936, rDf = 12, r² = 0.01, pw = 0.059 |
| Body mass gained (%) | χ² = 1.93, p = 0.381, rDf = 12, r² = 0.13, pw = 0.202 |
| Adiposity |  |
| Visceral fat (g/100g) | χ² = 61.94, p < 0.001, rDf = 12, r² = 0.84, pw > 0.999 |
| Mesenteric fat (g/100g) | χ² = 20.04, p < 0.001, rDf = 12, r² = 0.63, pw = 0.982 |
| Epididymal fat (g/100g) | χ² = 98.09, p < 0.001, rDf = 12, r² = 0.89, pw > 0.999 |
| Retroperitoneal fat (g/100g) | χ² = 44.43, p < 0.001, rDf = 12, r² = 0.79, pw > 0.999 |
| Serum |  |
| Glucose (mg/dL) | χ² = 19.39, p < 0.001, rDf = 12, r² = 0.62, pw =0.979 |
| Insulin (ng/mL) | χ² = 35.10, p < 0.001, rDf = 12, r² = 0.74, pw > 0.999 |
| HOMA-IR index | χ² = 23.93, p < 0.001, rDf = 12, r² = 0.67, pw =0.994 |
| Corticosterone (ng/mL) | χ² = 32.10, p < 0.001, rDf = 1.5, r² = 0.73, pw = 0.999 |
| Triacylglycerol (mg/dL) | χ² = 338.94, p < 0.001, rDf = 12, r² = 0.97, pw > 0.999 |
| Cholesterol (mg/dL) | χ² = 3.45, p = 0.178, rDf = 12, r² = 0.22, pw = 0.350 |
| HDL cholesterol (mg/dL) | χ² = 16.75, p < 0.001, rDf = 12, r² = 0.58, pw = 0.955 |
| n-HDL cholesterol (mg/dL) | χ² = 2.21, p = 0.330, rDf = 12, r² = 0.16, pw = 0.248 |
| Liver |  |
| Glycogen (mg/g) | χ² = 47.75, p < 0.001, rDf = 12, r² = 0.87, pw > 0.999 |
| Triacylglycerol (mg/100g) | χ² = 18.02, p < 0.001, rDf = 12, r² = 0.61, pw = 0.973 |
| Cholesterol (mg/100g) | χ² = 1.06, p = 0.587, rDf = 12, r² = 0.08, pw = 0.135 |
| HDL cholesterol (mg/100g) | χ² = 2.11, p = 0.348, rDf = 12, r² = 0.15, pw = 0.232 |
| n-HDL cholesterol (mg/100g) | χ² = 0.90, p = 0.637, rDf = 12, r² = 0.07, pw = 0.123 |
| (rDf) residual degrees of freedom; (pw) power. Samples size = 5/group | |

| **Supplementary table 2. Model information on single observation data parameters: inflammation and oxidative stress.** | |
| --- | --- |
| Dependant variable | Model information |
| Serum |  |
| IL6 | χ² = 0.17, p = 0.917, rDf = 12, r² = 0.01, pw = 0.059 |
| IL10 | χ² = 0.21, p = 0.898, rDf = 12, r² = 0.01, pw = 0.059 |
| TNFα | χ² = 13.56, p = 0.001, rDf = 12, r² = 0.53, pw = 0.908 |
| IL1β | χ² = 31.20, p < 0.001, rDf = 12, r² = 0.70, pw = 0.998 |
| Hypothalamus |  |
| IL6 | χ² = 19.87, p < 0.001, rDf = 12, r² = 0.62, pw = 0.979 |
| IL10 | χ² = 8.77, p = 0.012, rDf = 12, r² = 0.42, pw = 0.739 |
| TNFα | χ² = 12.36, p = 0.002, rDf = 12, r² = 0.51, pw = 0.883 |
| IL1β | χ² = 52.54, p < 0.001, rDf = 12, r² = 0.81, pw > 0.999 |
| Liver |  |
| IL6 | χ² = 14.04, p = 0.009, rDf = 12, r² = 0.54, pw = 0.929 |
| IL10 | χ² = 24.13, p < 0.001, rDf = 12, r² = 0.67, pw = 0. 995 |
| TNFα | χ² = 1.74, p = 0.420, rDf = 12, r² = 0.13, pw = 0.200 |
| IL1β | χ² = 1.69, p = 0.430, rDf = 12, r² = 0.12, pw = 0.196 |
| Hippocampus |  |
| IL6 | χ² = 18.82, p < 0.001, rDf = 12, r² = 0.61, pw = 0.973 |
| IL10 | χ² = 1.88, p = 0.390, rDf = 12, r² = 0.14, pw = 0.217 |
| TNFα | χ² = 19.04, p < 0.001, rDf = 12, r² = 0.62, pw = 0.979 |
| IL1β | χ² = 7.81, p = 0.020, rDf = 12, r² = 0.36, pw = 0.623 |
| Mesenteric fat |  |
| IL6 | χ² = 2.93, p = 0.231, rDf = 12, r² = 0.19, pw = 0.297 |
| IL10 | χ² = 10.397, p = 0.004, rDf = 12, r² = 0.48, pw = 0.841 |
| TNFα | χ² = 30.11, p < 0.001, rDf = 12, r² = 0.72, pw = 0.999 |
| IL1β | χ² = 9.41, p = 0.009, rDf = 12, r² = 0.43, pw = 0.757 |
| Hippocampus |  |
| SOD | χ² = 48.46, p < 0.001, rDf = 12, r² = 0.81, pw > 0.999 |
| CAT | χ² = 0.07, p = 0.954, rDf = 12, r² = 0.01, pw = 0.059 |
| MDA | χ² = 15.33, p < 0.001, rDf = 12, r² = 0.58, pw = 0.955 |
| CBP | χ² = 49.61, p < 0.001, rDf = 12, r² = 0.79, pw > 0.999 |
| (rDf) residual degrees of freedom; (pw) power. Samples size = 5/group | |

| **Supplementary table 3. Cytokines absolute concentrations.** | | | |
| --- | --- | --- | --- |
| Serum |  |  |  |
| IL6 | 105.921 ±20.317 | 97.767 ±32.846 | 99.498 ±39.961 |
| IL10 | 25.776 ±4.545 | 23.621 ±12.744 | 27.182 ±16.676 |
| TNFα | 21.536 ±1.012 | 22.541 ±0.724 | 23.665 ±0.969 |
| IL1β | 18.193 ±6.509 | 53.293 ±18.477 | 23.154 ±6.311 |
| Hypothalamus |  |  |  |
| IL6 | 0.991 ±0.085 | 1.04 ±0.129 | 1.388 ±0.236 |
| IL10 | 0.725 ±0.074 | 0.887 ±0.108 | 0.76 ±0.088 |
| TNFα | 2.904 ±0.105 | 3.161 ±0.219 | 2.868 ±0.078 |
| IL1β | 0.316 ±0.031 | 0.141 ±0.034 | 0.285 ±0.051 |
| Liver |  |  |  |
| IL6 | 5.058 ±1.049 | 5.927 ±1.301 | 3.586 ±0.446 |
| IL10 | 0.736 ±0.02 | 0.899 ±0.136 | 0.64 ±0.049 |
| TNFα | 2.328 ±0.235 | 2.402 ±0.083 | 2.279 ±0.061 |
| IL1β | 1.986 ±0.481 | 2.16 ±1.101 | 1.593 ±0.24 |
| Hippocampus |  |  |  |
| IL6 | 1.087 ±0.096 | 1.522 ±0.285 | 1.484 ±0.154 |
| IL10 | 0.899 ±0.05 | 0.943 ±0.19 | 1.019 ±0.146 |
| TNFα | 2.822 ±0.443 | 3.119 ±0.214 | 3.724 ±0.167 |
| IL1β | 0.516 ±0.176 | 0.356 ±0.156 | 0.675 ±0.188 |
| Mesenteric fat |  |  |  |
| IL6 | 4.624 ±1.95 | 3.328 ±0.585 | 4.103 ±1.086 |
| IL10 | 0.637 ±0.142 | 0.424 ±0.064 | 0.506 ±0.104 |
| TNFα | 2.441 ±0.123 | 2.306 ±0.103 | 2.751 ±0.165 |
| IL1β | 0.806 ±0.082 | 1.025 ±0.312 | 0.686 ±0.098 |
|  | | | |

| **Supplementary table 4. Model information on single observation data parameters: anxiety-like behaviour.** | |
| --- | --- |
| Dependant variable | Model information |
| Open field |  |
| Lines crossed | χ² = 28.01, p < 0.001, rDf = 12, r² = 0.70, pw = 0.999 |
| Time (Centre) | χ² = 12.32, p = 0.002, rDf = 12, r² = 0.52, pw = 0.892 |
| Lines crossed (peripheral zone) | χ² = 30.69, p < 0.001, rDf = 12, r² = 0.69, pw > 0.999 |
| Open Arms (EPM) |  |
| Entries (% total entries) | χ² = 26.73, p < 0.001, rDf = 12, r² = 0.69, pw = 0.997 |
| Time (% total time) | χ² = 13.82, p = 0.001, rDf = 12, r² = 0.54, pw = 0.919 |
| Distance (% total distance) | χ² = 27.93, p < 0.001, rDf = 12, r² = 0.70, pw = 0.998 |
| Average speed (m/s) | χ² = 0.05, p = 0.973, rDf = 12, r² = 0.00, pw = 0.054 |
| Immobility (s) | χ² = 3.98, p = 0.137, rDf = 12, r² = 0.25, pw = 0.414 |
| Enclosed Arms (EPM) |  |
| Entries (% total entries) | χ² = 1.31, p = 0.519, rDf = 12, r² = 0.10, pw = 0.160 |
| Time (% total time) | χ² = 4.02, p = 0.134, rDf = 12, r² = 0.25, pw = 0.405 |
| Distance (% total distance) | χ² = 4.69, p = 0.096, rDf = 12, r² = 0.28, pw = 0.463 |
| Average speed (m/s) | χ² = 8.67, p = 0.013, rDf = 12, r² = 0.42, pw = 0.754 |
| Immobility (s) | χ² = 9.28, p = 0.010, rDf = 12, r² = 0.44, pw = 0.784 |
| Centre (EPM) |  |
| Entries (% total entries) | χ² = 12.35, p = 0.002, rDf = 12, r² = 0.51, pw = 0.883 |
| Time (% total time) | χ² = 17.20, p < 0.001, rDf = 12, r² = 0.59, pw = 0.962 |
| Distance (% total distance) | χ² = 22.08, p < 0.001, rDf = 12, r² = 0.65, pw = 0.989 |
| Average speed (m/s) | χ² = 0.97, p = 0.617, rDf = 12, r² = 0.08, pw = 0.130 |
| Immobility (s) | χ² = 0.64, p = 0.726, rDf = 12, r² = 0.05, pw = 0.101 |
| Final third of the OA (EPM) |  |
| Entries (% open arms) | χ² = 11.64, p = 0.003, rDf = 12, r² = 0.49, pw = 0.855 |
| Time (% open arms) | χ² = 53.21, p < 0.001, rDf = 12, r² = 0.82, pw > 0.999 |
| Distance (% open arms) | χ² = 72.87, p < 0.001, rDf = 12, r² = 0.86, pw > 0.999 |
| Risk assessment |  |
| Protected head dips (s) | χ² = 8.48, p = 0.014, rDf = 12, r² = 0.41, pw = 0.744 |
| Unprotected head dips (s) | χ² = 16.02, p < 0.001, rDf = 12, r² = 0.57, pw = 0.957 |
| Unprotected head dips (% total head dips | χ² = 11.21, p = 0.004, rDf = 12, r² = 0.48, pw = 0.853 |
| Protected head dips (% protected time) | χ² = 14.58, p < 0.001, rDf = 12, r² = 0.53, pw = 0.928 |
| Unprotected head dips (% unprotected time) | χ² = 1.16, p = 0.561, rDf = 12, r² = 0.08, pw = 0.133 |
| Anxiety-like behaviour (PCA) | χ² = 20.07, p < 0.001, rDf = 12, r² = 0.63, pw = 0.985 |
| (rDf) residual degrees of freedom; (pw) power. Samples size = 5/group | |

| **Supplementary table 5. 95% Confidence interval for correlations between inflammation, oxidative stress, and the open arms parameters.** | | | | | | | | |
| --- | --- | --- | --- | --- | --- | --- | --- | --- |
|  | Serum Cort. | Serum TNFα | Hepatic IL6 | Ht IL6 | Hc TNF | M.FAT TNFα | Hc SOD | Hc MDA |
| Centre (OF) |  |  |  |  |  |  |  |  |
| Time (s) |  |  | 0.286 to 0.891 |  |  | −0.812 to −0.003 |  |  |
| Open Arms (EPM) |  |  |  |  |  |  |  |  |
| Entries (% total entries) | −0.931 to −0.489 | −0.861 to −0.163 |  | −0.892 to −0.290 | −0.813 to −0.003 | −0.918 to −0.416 | −0.903 to −0.342 | −0.818 to −0.019 |
| Time (% total time) | −0.886 to −0.266 |  | 0.336 to 0.902 | −0.894 to −0.299 |  |  |  |  |
| Distance (% total distance) | −0.882 to −0.249 |  | 0.227 to 0.877 | −0.936 to −0.520 |  | −0.841 to −0.092 | −0.913 to −0.391 |  |
| Final third of the OA (EPM) |  |  |  |  |  |  |  |  |
| Time (% open arms) | −0.874 to −0.216 | −0.890 to −0.281 |  | −0.896 to −0.312 | −0.899 to −0.325 | −0.836 to −0.078 | −0.899 to −0.325 | −0.834 to −0.069 |
| Distance (% open arms) | −0.917 to −0.411 |  | 0.513 to 0.935 | −0.929 to −0.476 | −0.847 to −0.113 | −0.889 to −0.276 | −0.943 to −0.561 | −0.876 to −0.225 |
| Unprotected head dips (EPM) | −0.906 to −0.357 | −0.832 to −0.063 | 0.152 to 0.858 | −0.950 to −0.603 |  | −0.879 to −0.234 | −0.863 to −0.173 |  |
| ANX (PCA) | 0.257 to 0.884 |  | −0.891 to −0.289 | 0.484 to 0.930 |  | 0.866 to 0.184 | 0.350 to 0.905 |  |
| Cort: corticosterone; Hc: hippocampal; Ht: hypothalamic; M.FAT: mesenteric fat; SOD: superoxide dismutase; MDA: malondialdehyde * indicates p < 0.05 | | | | | | | | |
